# Roughness Encoding in Human and Biomimetic Artificial Touch: Spatiotemporal Frequency Modulation and Structural Anisotropy of Fingerprints

**DOI:** 10.3390/s110605596

**Published:** 2011-05-26

**Authors:** Calogero Maria Oddo, Lucia Beccai, Johan Wessberg, Helena Backlund Wasling, Fabio Mattioli, Maria Chiara Carrozza

**Affiliations:** 1 The BioRobotics Institute, Scuola Superiore Sant’Anna, Polo Sant’Anna Valdera, Viale Rinaldo Piaggio 34, 56025 Pontedera, PI, Italy; E-Mail: chiara.carrozza@sssup.it (M.C.C.); 2 Center for Micro-BioRobotics@SSSA, Istituto Italiano di Tecnologia (IIT), Viale Rinaldo Piaggio 34, 56025 Pontedera, PI, Italy; E-Mails: lucia.beccai@iit.it (L.B.); fabio.mattioli@iit.it (F.M.); 3 Department of Physiology, University of Gothenburg, Medicinaregatan 11, SE-40530 Goteborg, Sweden; E-Mails: wessberg@physiol.gu.se (J.W.); helena.backlund@physiol.gu.se (H.B.W.)

**Keywords:** MEMS tactile sensor array, fingerprints, biomimetic fingertip, roughness encoding, artificial touch, mechanoreceptors, microneurography, human touch, biorobotics

## Abstract

The influence of fingerprints and their curvature in tactile sensing performance is investigated by comparative analysis of different design parameters in a biomimetic artificial fingertip, having straight or curved fingerprints. The strength in the encoding of the principal spatial period of ridged tactile stimuli (gratings) is evaluated by indenting and sliding the surfaces at controlled normal contact force and tangential sliding velocity, as a function of fingertip rotation along the indentation axis. Curved fingerprints guaranteed higher directional isotropy than straight fingerprints in the encoding of the principal frequency resulting from the ratio between the sliding velocity and the spatial periodicity of the grating. In parallel, human microneurography experiments were performed and a selection of results is included in this work in order to support the significance of the biorobotic study with the artificial tactile system.

## 1. Introduction

In this work we investigate the specific role of fingerprints in artificial touch by building tactile systems, inspired to the biological model, that embed artificial fingerprints with different geometries while leaving unchanged the other design features. This comparative approach is fundamental to define design parameters in developing bioinspired sensory systems but it could be also useful to provide hints and suggestions to develop experimental protocols and models in neurophysiology. BioRobotics offers the possibility to develop emulator of the human subjects [1], with different characteristics and design parameters, as in the work presented in this paper we selectively modify a specific parameter (the curvature of fingerprints) to evaluate the related effect. In order to support the significance of the artificial touch results, in parallel we present a selection of electrophysiological studies by means of microneurographic recordings of the activity of single, identified afferent units in the fingertips of healthy human volunteers [2].

We address the encoding of roughness, which is a major independent component of texture (together with softness, while stickiness is a minor component) [3,4]. Roughness is associated to the spatial modulation of the surface (*i.e*., spatial coarseness) and its perception is severely degraded in case of lack of tangential motion between the fingertip and the tactile stimuli (*i.e*., dynamic *vs*. static touch) [5,6].

The biomimetic fingertip experimented for artificial roughness encoding was designed and built by means of a MEMS based technological approach, integrating an array of microscale tactile sensors and polymeric fingerprint-like packaging. Biomimetism was achieved by reaching a density similar to the innervation of type I mechanoreceptors in humans (e.g., SAI have a density of about 70 units/cm^2^ [7]), by having a fine skin-like packaging layer above the MEMS sensors, as for the positioning of slowly adapting type I (SAI; Merkel) and rapidly adapting (RA; Meissner) units, and by mimicking the coarseness of human fingerprints (between-ridge distance typically comprised within 0.3 mm and 0.5 mm [8]).

To make a comparative analysis between the human subject and the artificial system, the same class of tactile stimuli is presented to both the biomimetic fingertip and to human subjects via dynamic passive touch protocols implemented through a mechatronic platform that can indent the stimuli to the fingertip and slide them in a smooth tangential fashion. We use periodic ridged stimuli (namely gratings, which can be considered as a kernel of more realistic polyharmonic surfaces used in various studies [9,10]) in order to show, in artificial touch, that the structure of fingerprints affects the directional isotropy in the encoding of the principal spatiotemporal frequency of stimuli. In this attempt we get inspiration from previous observations with monkey subjects providing evidence that gratings locally oriented parallel to the finger ridges elicit stronger response than tactile stimuli oriented along the orthogonal direction [11].

The working principle of our artificial touch system was explained in [12] and, coherently with other human or artificial touch studies [13–15], considers the spectral content of the mechanical vibrations elicited by textured surfaces. Briefly, when a relative motion occurs at finger-stimulus interface, provided that it is possible to estimate somehow the relative velocity *v*, the spatial period Δ*p_s_* of the grating is in inversely proportional relationship with the principal frequency *f_princ_* of the elicited mechanical vibration [16], such that:

(1) $$f_{\text{princ}}\;=\;\frac{v}{\Delta p_{s}}$$

The effectiveness in roughness encoding via dynamic artificial touch is in the capability to elicit such vibrations by stimulus-skin interface, by motion dynamics and by contact mechanics, and then to gather them via the sensing units located under the covering material [15,17–19].

Recently it has been asserted that human fingerprints contribute to the encoding of fine textures as they may perform spectral selection and amplification of tactile information in the frequency band, centered at about 250 Hz, of optimal sensitivity of Pacinian afferents [20]. In that work, Scheibert and colleagues, by experimenting an artificial tactile sensing technology, showed a principal frequency differing from Equation (1), since the spatial period Δ*p_f_* of fingerprints appeared (instead of Δ*p_s_*) in the dominant vibrations gathered by the tactile sensor, resulting in *f_princ_* = *v*/Δ*p_f_*. In our opinion, in [20] the dominance of finger skin geometry (Δ*p_f_*) on stimulus surface features (Δ*p_s_*) in the retrieved principal spectral component was: (1) activated by the used stimulus, whose edges were positioned randomly (white-noise 1D patterning, *i.e*., extremely polyharmonic), and (2) gathered thanks to the quite wide receptive field of the sensor due to the relatively thick 2 mm packaging layer (a relevant related analysis is provided in [21]) mimicking the positioning of deeply located (*i.e*., type II) Pacinian mechanoreceptors.

**Figure 1.**
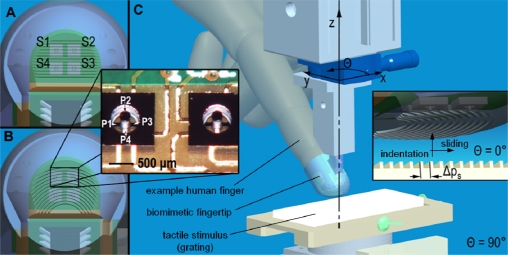
Design of the biomimetic fingertip integrating the rigid-flex board with 2 × 2 MEMS sensor array and readout electronics. Fingerprints embossed in the polymeric packaging had two curvatures. **Panel A** shows fingertip *a* design with straight fingerprints. **Panel B** shows fingertip *b* design with curved fingerprints. The inset shows two elements of the array of MEMS sensors. The piezoresistors (P1…P4) and the sensors (S1…S4) of the array are labeled according to the convention used in the text. **Panel C** shows a drawing of the experimental setup for indenting and sliding tactile stimuli in dynamic passive-touch experiments. An example human finger model is overlapped as a comparison to the developed biomimetic fingertip. The finger is rotated in steps of 10° along the *z*-axis (stimulus sliding across the distal phalanx in the depicted configuration, *i.e*., *θ* = 90°). The inset provides a close-up view of stimulus-artificial finger interface (stimulus sliding along the distal phalanx in the depicted configuration, *i.e*., *θ* = 0°).

As stated above, here the design of the tactile system gets inspiration from surface located human type I mechanoreceptors, we demonstrate a principal frequency modulation as from Equation (1), and in parallel we provide evidences of the same mechanism with RA human mechanoreceptors for fine and coarse gratings and with SAI for coarse gratings only (both RA and SAI are type I mechanoreceptors). Following this, we investigate in artificial touch how the shape of fingerprints affects the strength of principal frequency encoding by the embedded sensors. To this aim, we selectively change the morphology of the packaging encapsulating the sensor array (*i.e*., the curvature of fingerprints embedded in the polymeric skin-like outer layer as depicted in Figure 1(A,B)) and show the consequences on directional isotropy by means of experiments varying the reciprocal orientation between the artificial fingertip and the presented gratings (Figure 1(C)).

The manuscript is organized as follows. Section 2 presents the design of the experimented biomimetic fingertips, differing in the curvature of fingerprints, provides a brief description of the microneurography technique for human touch studies, and reports the experimental set-up and protocols. Section 3 introduces the data analysis techniques for human and artificial touch experiments. In Section 4 the experimental results are shown and discussed. Finally, the conclusions are provided in Section 5, together with insights on planned future work.

## 2. Materials

### 2.1. Biomimetic Fingertip

Four MEMS force micro-sensors [22,23] were integrated in a 2 × 2 array via flip-chip bonding on a rigid-flex board. Each sensor of the array was bonded on the rigid part on the corner of a square with a pitch Δ*X* of 2.36 mm, allowing the four tethers of each sensor to be suspended and free to flex under externally applied loads while the rigid support guaranteed stable mechanical bonding. Each sensor integrated four piezoresistors as sensing elements at the roots of the tethers forming a cross shape structure equipped with a mesa. This resulted in an array with 16 channels in total for transducing the mechanical interaction with external tactile stimuli. Inscribing each sensor in a square of area 5.57 mm^2^, a 0.72 channels/mm^2^ (16 channels/22.28 mm^2^) density was achieved, which mimics the SAI innervation density in humans (70 units/cm^2^) [7].

The 16 channels of the array were acquired by means of a high resolution (24 bit) Analog to Digital Converter (ADS1258, Texas Instruments, USA). The integration of the ADC onboard the fingertip allowed to reduce the amount of wires between the fingertip and the outer electronics, requiring power supply and a few digital communication channels only, and also guaranteed adequate signal-to-noise ratio due to the limited length of the connections routing analog signals. Data was sampled at 250 Hz per channel since such value was about one order of magnitude higher than the expected fundamental frequencies (as from Equation (1)); however, higher sampling frequencies are allowed by (1) increasing the overall conversion rate (this operation will affect the signal to noise ratio, but the achieved S/N levels guarantee that this is feasible) of the ADC lodged onto the fingertip, or (2) by reducing the number of converted channels (this operation will not affect S/N) without changing the overall conversion rate of the ADC. Acquired data was transmitted to a PC via Ethernet protocol by a soft-core processor (NiosII, Altera, USA) instantiated onboard a FPGA (Cyclone II, Altera, USA).

The rigid-flex board with MEMS sensor array and readout electronics was integrated in a rigid fingertip mimicking human anthropometry (Figure 1). The fingertip was designed for application to distal phalanxes of robotic hands being appropriate for grasping and manipulation tasks in anthropomorphic manner [24,25] and was fabricated with rapid prototyping resin via a 3D printer.

The packaging skin-like layer of the 2 × 2 array of MEMS sensors was introduced to have a similar function to the epidermal ridges of a human finger that can enhance deformation and frictional properties of the fingertip surface. Significant contributions in the simulative analysis [26,27] and artificial emulation [27,28] of fingerprints were given by Maeno and colleagues, showing that their structure increases the sensitivity in tactile activities with a major effect on surface located type I receptors. Therefore, fingerprints were included in the design of the proposed biomimetic fingertip considering that the epidermal ridges and grooves of an adult human have width in the 100–300 μm range, and the typical between-ridge distance is 400 μm [8,26,28].

The encapsulation was performed by means of soft polymeric packaging (Dragon Skin, Smooth-On, USA), having shore A 10 hardness and recovering its original form after a mechanical stimulation. The packaging material was poured directly on the fingertip by means of a mould that allowed to pattern the surface of the skin-like layer on top the sensor array.

Each single sensor of the array provides local information on the contact interaction at its interface with the surrounding polymeric packaging material, with the advantages of distributed tactile sensing [29]; in addition, our array of tactile sensors provides also directional information by means of the output readings from the four piezoresistors (implanted each at a root of a tether). Therefore, to have four outputs from each sensor increases the informative content on the stress state locally at each sensor site.

In order to investigate the role of the shape of fingerprints in texture encoding, two curvatures were designed (Figure 1(A,B)). In finger *a*, fingerprints were embossed with straight parallel ridges having between-ridge distance Δ*p_f_* set to 400 μm. Finger *b* had concentric fingerprints with groove and ridge widths as for prototype *a*, and the fingerprint passing from the center of the sensor array had curvature radius of 4.8 mm.

**Figure 2.**
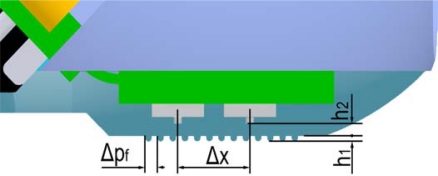
Cross section of the biomimetic fingertip, showing two sensors of the array and the structure and dimensions of fingerprints. The array pitch Δ*X* is 2.36 mm, the fingerprints have between-ridge distance Δ*p_f_* set to 400 μm, while their thickness *h_1_* is 170 μm. The thickness *h_2_* of the homogeneous packaging layer covering the sensor array is 600 μm.

As regards the thickness, here we used an artificial epidermal ridge with a height *h_1_* of 170 μm, while the thickness *h_2_* of the homogeneous packaging layer covering the sensor array was 600 μm (Figure 2); this resulted in sensing units being located quite close to the surface of the fingerpad, similarly to the positioning of type I human mechanoreceptors [26,28].

Preliminary load-unload tests with smooth flat surfaces were performed (not shown in this work) and showed that, for both the fingertip designs, the design of the packaging allows to reach at least up to 8 N normal and 4 N tangential forces without any damage to the encapsulated sensors.

### 2.2. Microneurography Technique for Human Touch Studies

Impulses of single tactile afferents in the left index and middle fingers were recorded using the microneurographic technique in 36 human healthy volunteers [2]. The subjects seated comfortably in a dentist’s chair, the left arm resting in a vacuum cast for stabilization and maximum comfort. Tungsten needle electrodes were inserted in the left median nerve, 8 cm above the elbow. The nerve signal was band-pass filtered at 200–4,000 Hz, sampled at 12.8 kHz together with analog data from the tactile stimulation mechatronic platform, and stored on a PC using the ZOOM/SC system developed at the Department of Physiology, Umeå University, Sweden. Recorded nerve impulses were inspected off-line on an expanded time scale using in-house software implemented in MATLAB (The Mathworks) and were accepted for subsequent analyses only if they could be validated as originating from a single afferent. Before running the experimental protocol, the units’ responses and receptive fields were explored using calibrated nylon filaments (von Frey hairs) and were classified as SAI, SAII, RA, or PC according to the adaptation of the response to sustained stimulation and size of the receptive field [30,31].

### 2.3. Experimental Set-Up and Protocol

The study focused on experimenting passive-touch protocols in which periodic ridged stimuli were indented (*z* direction, Figure 1(C)) and slided (*y* direction) on the human fingertip and on the two artificial fingertip prototypes (differing in the curvature of fingerprints).

**Figure 3.**
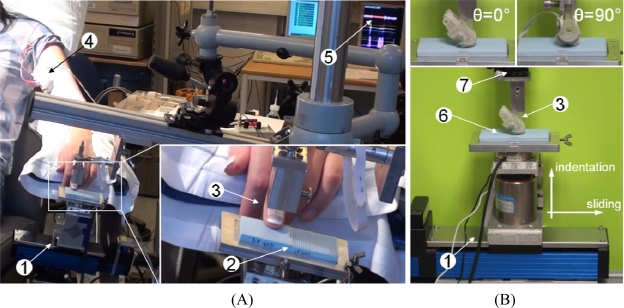
Experimental set-up in human (**Panel A**) and artificial (**Panel B**) touch experiments. 1: core mechatronic tactile stimulation platform; 2: pair of half-gratings; 3: human and biomimetic finger support; 4: first stage of microneurography electronics; 5: display with neural data for experiment monitoring; 6: single full-grating; 7: rotational stage with goniometer.

The passive-touch stimulation sequences were implemented by means of a mechatronic platform [32] with which repeatable experiments could be performed (Figures 1(C) and 3). This consisted in a 2 DoF system that could indent and slide textured stimuli to the fingertip. The system performed a feedback control on the normal contact force (*i.e*., indentation along the *z* direction) and a precise position/velocity control while recording the normal and tangential forces at finger-stimulus interface. The tactile stimulator could present stimuli to the fingertip without being affected by spurious vibrations and covering a range of forces and movement velocities as those that would be used by humans while exploring textures [4,6], *i.e*., at least from 100 mN up to 5 N indentation force and up to 150 mm/s tangential sliding velocity. The same core mechatronic tactile stimulator was used for human (Figure 3(A)) and artificial touch (Figure 3(B)) experiments. Periodic ridged surfaces (gratings), fabricated from tufset rigid polyurethane thermosetting plastics material, were tested as tactile stimuli in both human and artificial touch experiments.

#### Human Touch Experiments

During microneurographic experiments, gratings measuring 32 mm × 35 mm each and mounted in pairs on changeable plates (Figures 1(C) and 3(A)) were experimented. The experiments were performed with gratings having spatial period Δ*p_s_* (defined in the inset of Figure 1(C)) between 280 μm and 1,920 μm, with normal contact force set to 100 mN, 200 mN, 400 mN or 800 mN, sliding distance of 24 mm and velocity from 5 mm/s up to 40 mm/s among different sessions. Overall, 10 RA, five SAI and three SAII single afferent units were successfully recorded in the fingerpad with different grating spatial periods, while the platform applied the sliding motions, repeated in runs of 12, across the distal phalanx (*i.e*., according to the reference frame in Figure 1(C), *θ* was fixed at 90°). According to previous studies [33,34], the finger-stimulus contact area monotonically increases (positive first derivative), with decreasing slope (negative second derivative), with respect to the contact force; such previous studies report a contact area lower than 1 cm^2^ at the maximum force loads used in the human touch studies presented in this work. However, the preparatory session with calibrated Von Frey hairs, to characterize the locations and receptive fields of the recorded human tactile units, guaranteed the contact zone with the grating to cover the receptive field of the unit which was recorded during each experimental session. Human experiments were conducted according the Declaration of Helsinki and the ethics committee at the University of Gothenburg approved the study.

#### Artificial Touch Experiments

The artificial fingertip was fixed to the tactile stimulator by means of a mechanical support, interfaced to a rotational stage with goniometer (07 TRT 508, Melles Griot, USA, depicted in blue in Figure 1(C) and labeled as 7 in the picture of Figure 3(B)), that kept the fingertip surface parallel to the stimulus surface. As for human touch experiments, gratings were used as tactile stimuli. Each grating measured 32 mm × 75 mm. Therefore, the experimented gratings had approximately double length than those used with human subjects and each was separately (rather than in half-pairs as for human touch experiments) mounted on changeable plates. This choice was operated to test a higher sliding distance for each stimulus, since in artificial touch there is no relevant constraint to acquire as many data as possible within each session, oppositely to the looming risk of missing the nerve signal during human microneurography experiments. Two surfaces were evaluated, with spatial periods Δ*p_s_* (defined in the inset of Figure 1(C)) of 360 and 440 μm. The indentation force was 200 mN, which is one of the values used in the ongoing human touch study and is within the range used by humans during tactile exploratory tasks [6]; the force level was not varied in this work since related previous artificial touch studies by our group [12,22] showed that a modulation of the contact force in the 100 mN–1 N range resulted in a principal frequency being coherent with Equation (1). The velocity was 10 mm/s, which is the lower boundary of the range of exploratory velocities typically used by humans [35], and was not varied in this work since previous studies [12,22,36] already showed (up to 48 mm/s, in [12]) that a change in velocity coherently modulates the principal frequency according to Equation (1). A more comprehensive investigation is left to future studies, which could investigate the effect on amplitude modulation at the expected principal frequency due to variations in the indentation force or in the sliding velocity.

**Figure 4.**
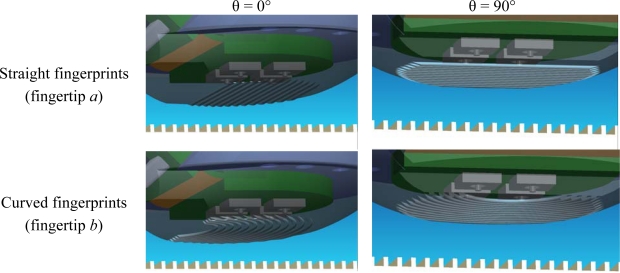
Protocol for the artificial touch experiments: the two biomimetic fingertip prototypes, differing in the curvature of fingerprints, were rotated in steps of 10° from *θ* = 0° (stimulus sliding along the distal phalanx) to *θ* = 90° (stimulus sliding across the distal phalanx).

According to the experimental protocol, the two artificial fingertip prototypes *a* (straight fingerprints, Figure 4-top) and *b* (curved fingerprints, Figure 4-bottom) were evaluated by rotating them from *θ* = 0° (stimulus sliding along the distal phalanx, Figure 4-left) to *θ* = 90° (stimulus sliding across the distal phalanx, Figure 4-right) in steps of 10°, thus indenting and sliding the ridged stimuli with ten different fingertip orientations. After enabling data acquisition, the stimulator applied the 200 mN feedback-regulated indentation force to the biomimetic fingertip. Subsequently, the stimulus was stroked at 10 mm/s, with the fingertip oriented along the selected direction, while maintaining enabled the force feedback controller. The sliding distance was set to 60 mm, providing a dynamic stimulation (corresponding to surface-fingertip tangential relative motion) for a duration of 6 s. Before commanding the stimulator to unload the fingertip, a steady state was applied, with contact force held at the 200 mN reference value.

In each experimental run a *combination* of stimulus (having spatial period Δ*p_s_*) and of fingertip orientation angle (*θ*) was used with a fingertip prototype (*a* or *b*). Six runs were repeated per *combination*, resulting in 240 runs in total (2 fingertip designs × 2 gratings × 10 angles × 6 repetitions).

The velocity and the start and stop absolute positions along the sliding direction were not varied among different *combinations* or among repeated runs with the same *combination*.

## 3. Methods

### 3.1. Human Touch Data Analysis

Neural data was at first inspected and processed in time domain to identify neural events. Then, the identified spikes were analyzed by obtaining spectra of the spike trains from single afferent units using the approach for frequency domain analysis for point processes [37]. For frequency domain analysis, 1.0 second data windows without overlap were used, and spectra were obtained from disjoint windows for all the available data for a specific stimulation condition (*i.e*., *combination* of sliding velocity, surface type, and normal force). Quantitative analysis was then performed across all the acquired data to evaluate whether or not a specific class of tactile units presented a spectrum with a principal frequency according to Equation (1).

### 3.2. Artificial Touch Data Analysis

After a preliminary graphical inspection of data from the biomimetic fingertip in time domain, the core analysis was operated in time-frequency domain via Short Time Fourier Transform (STFT) and in frequency domain via Fast Fourier Transform (FFT) over 1,024 samples (4.096 s of data at 250 Hz) in the middle of the stimulus sliding phase.

A recent study in active touch showed outstanding repeatability of experimental data by means of the fingertip having curved fingerprints [36]. Those previous results allowed us to directly consider here the aggregated results rather than data on a single run basis, also because real-time is not targeted in this work. Therefore, the six repeated runs per *combination* were aligned off-line and averaged channel by channel, enhancing their statistical significance and emphasizing their informative content.

For quantitative analysis of biomimetic fingertip experimental results, the principal spectral component *f_peak_* is retrieved from outputs of the sensor array in order to evaluate the matching with the expected frequency *f_princ_* (Equation (1)):

(2) $$f_{\text{peak}}\;=\;{\text{arg}\;\text{max}}_{f>f_{0}}\;(|\text{FFT}(\text{PiSj})|)$$

where the *argmax* function returns the frequency, provided that it is higher than the lower boundary *f_0_*, carrying the highest power in the FFT of the output data from piezoresistor *Pi* of sensor *Sj*. In the considered analysis, *f_0_* was set to 2.5 Hz, so to discard the very low-frequency spectral components.

## 4. Results and Discussion

**Figure 5.**
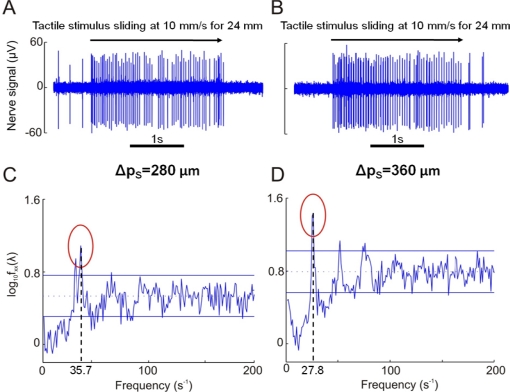
**Panels A** and **B** show microneurographic recordings from human single tactile RA afferents in the fingertips during stimulation as in Figures 1(C) and 3(A); a 10 mm/s sliding motion was applied across the distal phalanx. **Panels C** and **D** show spectral analysis of the nerve discharge trains from 12 repeated stimulus runs for the units shown in A and B. Grating spatial periodicity Δ*p_s_* is 280 μm in A and C, 360 μm in B and D. Principal frequencies resulting from Equation (1) according to the specific *combination* of grating spatial periodicity Δ*p_s_* and sliding velocity *v* are 35.7 and 27.8 Hz for Panels C and D, respectively, with meaningful coherence with the depicted experimental results. Horizontal lines in C and D show p < 0.01 confidence limits for significant frequency modulation.

The presented results from human microneurography experiments (Figure 5) provide evidence of modulation of single unit firing according to Equation (1). The experiments with the biomimetic fingertip show such modulation as well, confirming significance of the presented artificial touch investigation, carried out in parallel to human touch studies. A compared analysis of results from biomimetic fingertip *b* with curved fingerprints (Figures 7, 8(E–H), 9(B) and 10(B)), and from biomimetic fingertip *a* with straight fingerprints (Figures 8(A–D), 9(A) and 10(A)) shows that the curvature of fingerprints has consequences on isotropy in the encoding of roughness while rotating the fingertip.

### 4.1. Human Touch

Figure 5 shows sample nerve recordings, gathered from the median nerve above the elbow using the microneurographic technique [2], from a human RA receptor during stimulus sliding motion across the distal phalanx (*i.e*., *θ* = 90°). The subject’s fingerprint at the location of the depicted RA unit has a tangent oriented at approximately 46° from the direction parallel to the ridges of the gratings (=44° degrees from the direction of the sliding motion). Spectral analysis of the nerve discharge patterns [37] showed significant modulation at the frequency determined by the stimulus spatial period Δ*p_s_*, according to Equation (1). A similar relationship depending on the stimulus spatial period was observed in the activity of single human mechanoreceptors with receptive fields in the finger tips of the second and third fingers. Particularly, for the tested gratings in the 280–520 μm spatial period range, this frequency-locked modulation was for 8 of 9 RA afferents units where this was tested, but not in any of the SAI units (n = 5). For gratings in the 1,600–1,920 μm spatial period range, it was observed in all of the tested RA and SAI units (n = 7 and 5, respectively; 10–20 mm/s sliding velocity). The smaller peak at 30.8 Hz in Figure 5(C) reflects a slight periodic modulation of unit firing that is uncorrelated with the periodicities in the mechanical stimulus. Moreover, it should be noted that the average discharge rates of single tactile afferents never directly reflected the spatial periods of the stimuli. As an example, average discharge rate was 40 Hz for the unit in Figure 5(A), and 55.5 Hz for the unit in Figure 5(B). Thus, there was no 1:1 (or higher order) locking of the nerve discharges, but the spatial periodicity were reflected as a frequency modulation (Equation (1)) of the discharge patterns.

### 4.2. Artificial Touch

**Figure 6.**
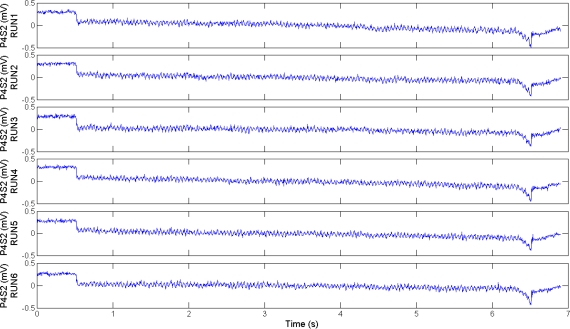
Outputs from P4S2 of the fingertip *b* with curved fingerprints, over six runs under the same experimental conditions (Δ*p_s_* = 440 μm and *θ* = 0°), showing high repeatability of experimental data.

Figure 6 shows the output signal from a channel of the tactile array over six runs recorded under the same experimental conditions, in order to assess high repeatability of data. Cursory analysis of Figure 6 confirms high similarity among the plots. As a further quantitative assessment, Table 1 presents the correlation indexes calculated over all the pairs of the runs plotted in Figure 6. All the correlation values reported in Table 1 are close to 1, confirming high repeatability. Similar repeatable results were obtained from the other channels of the array, for both the fingerprint designs and for all the experimental combinations (*θ* and Δ*p_s_* values). High repeatability allowed to perform the averaging operation for all the following experimental results, as detailed in Section 3.2, in order to provide significant information by means of data recorded under multiple runs.

**Figure 7.**
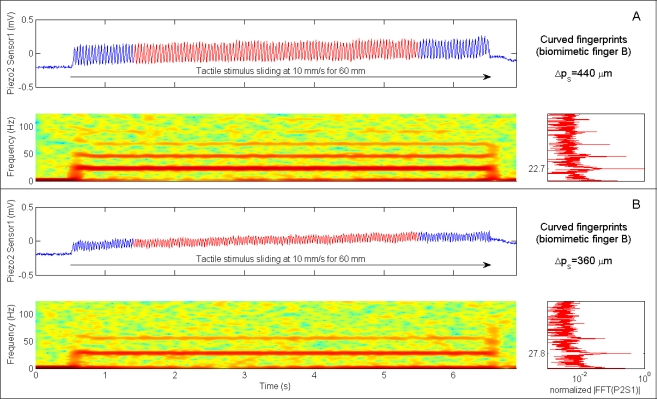
Encoding of stimulus spatial period Δ*p_s_* in either time, frequency and time-frequency domains. Data belongs to Piezoresistor 2 of Sensor 1 of the biomimetic fingertip and was acquired while sliding at 10 mm/s (200 mN indentation force) the 440 μm (**Panel A**) and 360 μm (**Panel B**) periodic stimuli over the biomimetic fingertip with curved fingerprints (shown in Figure 1(B)). According to Equation (1), the expected principal frequency was 22.7 Hz (A) or 27.8 Hz (B). The rotation of the fingertip was 10° with respect to the stimulus sliding direction (reference frame shown in Figure 1(C)).

**Figure 8.**
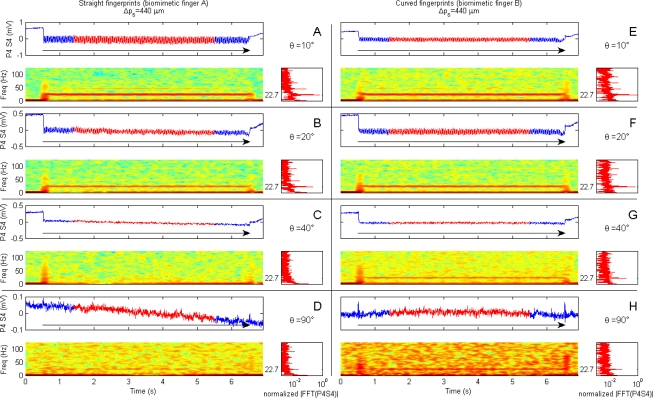
Encoding of stimulus spatial period Δ*p_s_* as a function of biomimetic fingertip rotation *θ* for both the prototypes with straight and curved fingerprints. Data belongs to Piezoresistor 4 of Sensor 4 of the biomimetic finger and was acquired while sliding at 10 mm/s (200 mN indentation force) the 440 μm periodic stimulus over the biomimetic finger with straight fingerprints (**Panels A** to **D**) and with curved fingerprints (**Panels E** to **H**). According to Equation (1), the expected principal frequency was 22.7 Hz. A description of each row of the subplots is provided within Figure 7. The plotted results are obtained by rotating the finger of an angle *θ* set to 10° (**A**, **E**), 20° (**B**, **F**), 40° (**C**, **G**) and 90° (**D**, **H**) with respect to the stimulus sliding direction.

In Figures 7 and 8 time domain data from single channels (Piezoresistor 2 of Sensor 1 and Piezoresistor 4 of Sensor 4, respectively) of the experimented biomimetic fingertip designs is plotted above the related STFT. The insets on the right of the STFT plots show the spectra obtained by applying a FFT to the single channel data highlighted in red in the time domain plots.

Particularly, Figure 7(A,B) shows time domain traces from Piezoresistor 2 of Sensor 1 in fingertip *b* (see Figure 1 for the labeling of sensors of the tactile array) during stimulation with 360 μm and 440 μm regular gratings rotated at an angle *θ* = 10°. The periodic patterns at 27.8 Hz (360 μm grating) and at 22.7 Hz (440 μm grating) associated to the spatial periodicity of tactile stimuli are clearly visible either in time (vibrational component), in frequency (dominant peak in the FFT, marked with a dotted line) and in time-frequency (red region, marked with a dotted line in the STFT) domains. Since the sliding velocity remains constant in the performed experiments, the dominant frequency of the vibrations elicited by the tactile stimulus is proportional to the inverse of the spatial period of the grating (Equation (1)), while the intensity of the vibrations increases with the spatial period. Both these effects appear to be coherent with [13], where the mechanical vibrations recorded in the fingertip of human subjects are shown to scale down in peak frequency and to increase in peak-to-peak amplitude while increasing the spatial period.

The relevance of the dynamic stimulation phase (*i.e*., the dataset corresponding to surface-fingertip tangential relative motion) to extract vibrational patterns which are correlated to the stimulus surface features is confirmed by the STFT spectrograms depicted below the time domain plots, which show a sudden frequency step at the onset of the stimulus sliding motion. The spectral pattern remains stable while the periodic grating is stroked at constant velocity. More importantly, as confirmed by the FFT spectra, the frequency peak corresponds to the expected value depending on the applied stimulus according to Equation (1), *i.e*., 27.8 Hz for the 360 μm surface and 22.7 Hz for the 440 μm one. Significantly, this artificial vibrational roughness encoding is coherent with the microneurography results in humans, according to the findings reported in Section 3.1 and to previous studies with monkeys [38].

A comparison between the A–D and the E–H panels in Figure 8 shows the effect of the curvature of fingerprints in the encoding of stimulus spatial features with respect to the rotation of the biomimetic fingertip. The four rows show results for *θ* = 10°, *θ* = 20°, *θ* = 40° and *θ* = 90°. There is higher isotropy with the curved fingerprints than with the straight ones, which have a strongly preferred direction when the sliding is closer to the direction along the distal phalanx (*i.e*., across the fingerprints). As shown in Figure 8(A), with straight fingerprints the vibratory patterns are noticeable either in time and frequency domains for *θ* = 10°, while those patterns are considerably reduced and masked by the other spectral components when the fingertip is rotated (Figure 8(B–D)) so to have a sliding oriented closer to the direction across the distal phalanx (*i.e*., along the fingerprints).

**Figure 9.**
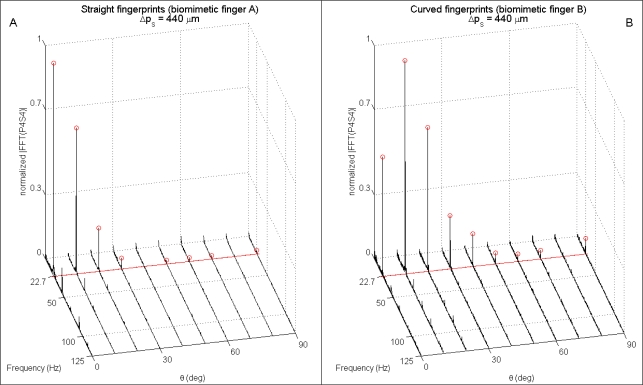
Single-sided normalized amplitude spectra as a function of the rotation of the biomimetic fingertip with straight (**Panel A**) and curved (**Panel B**) fingerprints. Data is related to 4.096 s subsets gathered from Piezoresistor 4 of Sensor 4 (P4S4) while the stimulus was indented and rubbed tangentially to the finger. Normal stimulus-fingertip contact force was set to 200 mN, while the sliding velocity was 10 mm/s. According to Equation (1), the expected principal frequency (marked with a red straight line) was 22.7 Hz. The red circles highlight the correctly identified (by applying Equation (2)) peak frequency per each stimulation *combination*. Higher isotropy as a function of the rotation angle is appreciated with the fingertip having curved fingerprints.

**Figure 10.**
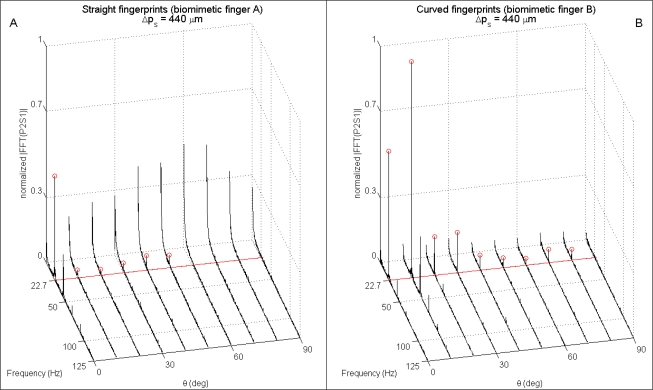
Single-sided normalized amplitude spectra as a function of the rotation of the biomimetic fingertip with straight (**Panel A**) and curved (**Panel B**) fingerprints. Data is related to 4.096 s subsets gathered from Piezoresistor 2 of Sensor 1 (P4S4) while the stimulus was indented and rubbed tangentially to the finger.

Extended analysis of the spectrum of readings from both the biomimetic finger designs as a function of the rotation angle *θ* brings evidence of the higher anisotropy anticipated above for straight fingerprints (Figure 9(A) compared to Figure 9(B) and Figure 10(A) compared to Figure 10(B)). Within the plots shown in Figures 9 and 10, the expected (Equation (1)) principal frequency is represented by a straight red line, while the peak arising in the frequency domain for output *PiSj* (*i^th^* piezoresistor of *j^th^* sensor, according to Figure 1) is detected by applying Equation (2) and the correctly identified ones are marked with red circles in the figures.

It is significant to point out that, differing from Figure 9(A) (straight fingerprints), in Figure 9(B) (curved fingerprints) the peak is not at *θ* = 0° but at *θ* = 10°. This is a consequence of the curvature of fingerprints, which affects the sensitivity of the packaged system in tradeoff with the preferred direction of Piezoresistor P4, hence widening the region of effective roughness encoding as a function of the finger rotation angle *θ*: the tangent to the curved fingerprints at the location of Sensor S4 is orthogonal to the *x* axis sliding motion direction (and contemporarily parallel to ridges of the grating) when *θ* = 17.4° (*i.e*., >10°), while piezoresistor P4 shows its maximum sensitivity [23,39] to tangential loads (reaching the sensor through the packaging material) being oriented along the direction of its tether, which is aligned with the *x* axis when *θ* = 0° (*i.e*., <10°).

Considerations similar to those reported above for Figure 9 apply to Figure 10 as well, which depicts spectral data from P2S1 as a function of the rotation angle *θ*. The tangent to the curved fingerprints at the location of Sensor S1 is orthogonal to the *x* axis sliding motion direction (and contemporarily parallel to ridges of the grating) when *θ* = 10.9°, which is lower than the related value for S4; as a consequence, in Figure 10(B) the peak at *θ* = 10° appear to be more marked than the one at *θ* = 20°, if compared with the same pair of peaks in Figure 9(B).

For all the channels of the tactile array, the design with straight fingerprints guaranteed an absolute error Δ*f* = |*f_princ_* − *f_peak_*| in principal frequency estimation (via Equation (2)) lower than 0.15 Hz for 75% of all the experimental *combinations*, while the percentage raised to 82.5% for the design with curved fingerprints.

## 5. Conclusions and Future Work

The presented results provide evidence that the stimulus spatial features are encoded in the spectral content (*i.e*., the principal frequency for a periodic grating) of the firing pattern in human mechanoreceptors and of the outputs of the developed biomimetic artificial fingertip. It is notable that in human touch this was observed for all the tested gratings in a large proportion of recorded RA human mechanoreceptor afferents and that in artificial touch the same roughness encoding mechanism, based on Equation (1), was fully demonstrated. In this work, the observed peak frequency values were at the expected values depending on the tested stimulus spatial period and constant sliding velocity tangential to the fingerpad, while the shape of the fingerprints was shown to have an effect on the possibility to promote and sense such vibrations, not in shifting the peak values on the frequency axis. The results presented here with simple gratings appear to go in the direction of those with more complex surfaces presented in [9] (e.g., see Figure 4 of [9]), since in such work the mechanical vibrations were found to have spectra repeatably related to the surfaces which were experimented with different subjects (therefore, having different fingerprints one to the other) at constant finger-stimulus relative velocity.

The experimental analysis of the artificial fingertip suggests that the structural anisotropy of fingerprints, due to their shape, has a major role in determining the level of anisotropy in the encoding of spatial features of tactile stimuli. The sensory systems with straight fingerprints embedded in the skin-like packaging had noticeably higher directional preference, while higher isotropy was observed with curved ones. The obtained results provide inputs for the design of artificial sensory systems to best encode textural features in case that the target application has or has not a preferred direction for the finger-stimulus relative motion.

Bensmaïa and colleagues raised the open question whether the anisotropy observed in humans is related to the structural anisotropy of the skin or to afferent branching at neural level [40]. In this work the experimented biomimetic artificial fingertips differed in the packaging skin-like layer design only; moreover, the anisotropy was observed on a channel by channel basis, not only as an aggregated effect among different outputs of the array. Therefore, from a robotic point of view the presented results agree with the hypothesis according to which the directional anisotropy is affected by the structure of fingerprints. Starting from these initial results, investigations on a potential concurrent role of afferent branching at neural level may be addressed in future by performing artificial touch experiments in accordance with human touch protocols, while also recording from afferent units by means of the microneurography technique and analyzing the firing modulation as a function of the stimulus sliding direction.

Finally, moving from the observation that humans appear to be able to discriminate tactile stimuli in a wide velocity range (from a few mm/s up to more than a hundred of mm/s) without any significant velocity induced effect on perceived roughness [35,41], future research via parallel artificial and human touch experiments will investigate a possible mechanism for removing the effect of velocity from Equation (1). This work has not addressed the real time discrimination of surfaces neither considered a time-varying velocity during the sliding motion of the tactile stimuli. Constant velocity was used to obtain stationary spectra in the frequency domain, which were analyzed to evaluate the role of the curvature of fingerprints in modulating the strength in the encoding of spatial wavelengths of tactile stimuli. To go towards real time discrimination feasibility under unconstrained non-constant sliding velocities, we will investigate the possibility for our artificial tactile system to implement the hypothetical human model based on coincidence detection of neural spikes, which was discussed in [42,43]. As a matter of fact, we believe that such model is promising in artificial touch as well, since a suitable approach should consider phase relationships between outputs from adjacent sensors of the array (as briefly anticipated in [36]), so to establish a spatio-temporal surface discrimination method, rather than spatial (*i.e*., taking into account static stimulus representation by distributed sensor units) or temporal (*i.e*., taking into account the vibrational stimulus representation by single sensor units) only.

## Figures and Tables

**Table 1.** Correlation indexes for all the pairs of experimental runs shown in Figure 6.

		**RUNS**					
		**1**	**2**	**3**	**4**	**5**	**6**
**RUNS**	**1**	1.00	0.94	0.92	0.95	0.94	0.93
	**2**	0.94	1.00	0.95	0.95	0.95	0.94
	**3**	0.92	0.95	1.00	0.94	0.94	0.94
	**4**	0.95	0.95	0.94	1.00	0.96	0.94
	**5**	0.94	0.95	0.94	0.96	1.00	0.94
	**6**	0.93	0.94	0.94	0.94	0.94	1.00
